# A phase I trial of DNA vaccination with a plasmid expressing prostate-specific antigen in patients with hormone-refractory prostate cancer

**DOI:** 10.1038/sj.bjc.6602019

**Published:** 2004-07-27

**Authors:** M Pavlenko, A-K Roos, A Lundqvist, A Palmborg, A M Miller, V Ozenci, B Bergman, L Egevad, M Hellström, R Kiessling, G Masucci, P Wersäll, S Nilsson, P Pisa

**Affiliations:** 1 Immune and Gene Therapy Laboratory, Cancer Center Karolinska, R8:01, Karolinska Institute, S-171 76 Stockholm, Sweden; 2 Department of Urology, Karolinska University Hospital, 171 76 Stockholm, Sweden; 3 Department of Pathology, 171 76 Karolinska University Hospital, Stockholm, Sweden; 4 Department of Oncology, 171 76 Karolinska University Hospital, Stockholm, Sweden

**Keywords:** prostate cancer, prostate-specific antigen, DNA vaccine, immune response, IFN-gamma, immunotherapy

## Abstract

Prostate-specific antigen (PSA) is a serine protease secreted at low levels by normal luminal epithelial cells of the prostate and in significantly higher levels by prostate cancer cells. Therefore, PSA is a potential target for various immunotherapeutical approaches against prostate cancer. DNA vaccination has been investigated as immunotherapy for infectious diseases in patients and for specific treatment of cancer in certain animal models. In animal studies, we have demonstrated that vaccination with plasmid vector pVAX/PSA results in PSA-specific cellular response and protection against tumour challenge. The purpose of the trial was to evaluate the safety, feasibility and biological efficacy of pVAX/PSA vaccine in the clinic. A phase I trial of pVAX/PSA, together with cytokine granulocyte/macrophage-colony stimulating factor (GM-CSF) (Molgramostim) and IL-2 (Aldesleukin) as vaccine adjuvants, was carried out in patients with hormone-refractory prostate cancer. To evaluate the biologically active dose, the vaccine was administered during five cycles in doses of 100, 300 and 900 *μ*g, with three patients in each cohort. Eight patients were evaluable. A PSA-specific cellular immune response, measured by IFN-*γ* production against recombinant PSA protein, and a rise in anti-PSA IgG were detected in two of three patients after vaccination in the highest dose cohort. A decrease in the slope of PSA was observed in the two patients exhibiting IFN-*γ* production to PSA. No adverse effects (WHO grade >2) were observed in any dose cohort. We demonstrate that DNA vaccination with a PSA-coding plasmid vector, given with GM-CSF and IL-2 to patients with prostate cancer, is safe and in doses of 900 *μ*g the vaccine can induce cellular and humoral immune responses against PSA protein.

Prostate cancer is the second most commonly diagnosed cancer and the fourth leading cause of cancer-related death in men in the developed countries worldwide (GLOBOCAN 2000, 2001). Effective curative treatment modalities are debilitating, and are only currently available for localised disease. In hormone-refractory prostate cancer, no agent has been shown to prolong survival beyond approximately 1 year. Tumour immunology is entering a new era due to the advances in our understanding of the immune network, and the identification of tumour antigens that can be targeted by therapeutic manipulation of the immune system. With regard to immunotherapy of prostate cancer, several prostatic tissue-confined antigens have been described, such as prostate-specific antigen (PSA), prostate-specific membrane antigen (PSMA), prostate acid phosphatase (PAP) and prostate secretory protein-94 (PSP94).

Prostate-specific antigen is a serine protease of the human glandular kallikrein family restricted to higher primates (Riegman *et al*, 1989). It is widely used in the early diagnosis and monitoring of prostate cancer (Catalona *et al*, 1991). The gene encoding the PSA protein is located on chromosome 19. Its androgen-regulated transcription results in the biosynthesis of a 261 amino-acid PSA precursor. By proteolytic cleavage, a 237 single chain form is secreted into the seminal fluid by the columnar epithelial cells. The PSA levels in seminal fluid are one million-fold higher than in serum. Normal PSA levels in the serum in males are below 4 ng ml^−1^. Far lower PSA expression has also been reported in other tissues, among others the female breast (Diamandis and Yu, 1995).

Different formulations of PSA have been used in several early clinical trials, demonstrating the possibility to induce immune responses against a self-antigen, such as PSA, using active immunisation. PSA has been formulated as recombinant protein in liposomes (Meidenbauer *et al*, 2000), in recombinant vaccinia virus (Eder *et al*, 2000), as mRNA in autologous DC (Heiser *et al*, 2002) or HLA-A24-associated peptides (Noguchi *et al*, 2003).

DNA vaccines represent the latest development in vaccination strategies (Ulmer *et al*, 1993). Direct introduction of plasmid DNA into the cells of living hosts (by gene gun, or needle injection) leads to the generation of both humoral and cellular immune responses and protective immunity. Some of the advantages of DNA vaccination are: specificity, nonimmunogenicity of the vector, mammalian post-translational modification, stability, safety, cost advantage and generic manufacturing. A serious disadvantage is the relatively poor immune response induced with plasmid DNA as compared to traditional vaccines (Liu, 2003) and therefore combinations with various adjuvants are currently tested. Based on published studies showing enhancement of DNA vaccines using interleukin-2 (IL-2) and granulocyte/macrophage-colony stimulating factor (GM-CSF) (Mincheff *et al*, 2000; Parmiani *et al*, 2000), and our preclinical results (unpublished), these two cytokines were chosen as adjuvants.

Clinical trials demonstrated that DNA vaccines coding for viral antigens induce significant levels of protein production (Liu, 2003), and evoke CD4^+^, CD8^+^ and antibody responses in non-cancer-bearing subjects. It is important to realise, however, that immune responses to microbial vaccines in healthy individuals are not comparable to immune responses to cancer vaccines in cancer patients (Velders *et al*, 1998). At present, little is known as to how the host-antigenic tolerance to tumour antigens in patients with active cancer interferes with DNA-vaccine-induced responses. In this respect, studies in animal models have severe limitations in reflecting the clinical situation (Lollini and Forni, 2002). The few published trials with DNA cancer vaccines give contradictory results (Timmerman *et al*, 2002; Rosenberg *et al*, 2003; Tagawa *et al*, 2003).

Since a naked DNA-vaccine coding for PSA has not been previously tested in humans, we performed a phase I trial to evaluate its adverse effects and determine the possible induction of PSA-specific immune response.

## MATERIALS AND METHODS

### Study rational and objectives

The aim of this phase I trial was to evaluate the safety and feasibility, and to determine the biologically effective dose of a DNA vaccine.

### Patient eligibility

The trial was approved by the Institutional Ethics Committee in accordance with the principles of the Declaration of Helsinki and the Swedish Medical Product Agency.

Nine patients with advanced prostatic adenocarcinoma were enrolled after informed, signed consent into the trial. Patients were diagnosed by fine-needle aspiration cytology and in one patient the diagnosis was obtained using core-biopsy. The cytology specimens were graded according to a three-tier system. The core-biopsy was reviewed and graded according to the Gleason system. Patients were required to have evidence of progressive disease determined by either new bone or soft tissue lesions or rising PSA level on three consecutive occasions despite androgen deprivation (and if applicable, antiandrogen withdrawal). Patients had to have the Eastern Cooperative Oncology Group performance status of 2 or less with an estimated life expectancy of at least 6 months. They were assessed as unlikely to require chemotherapy, radiation therapy or administration of corticosteroids (corresponding to >400 *μ*g day^−1^ of Bunesonide) for at least 6 months. Exclusion criteria included manipulation of hormonal therapy, radiation or chemotherapy within 10 weeks prior to inclusion, clinical evidence of central nervous system (CNS) metastases, active condition of allergy or autoimmune disease, noncutaneous malignant process, current or history of immunodeficiency, including previous splenectomy or irradiation of the spleen.

### Study design

**Figure 1:**
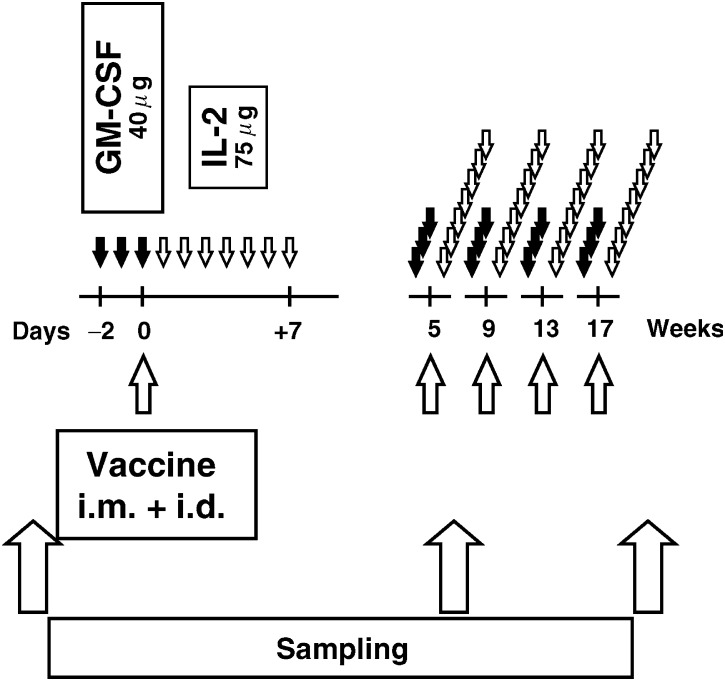
Flow chart of clinical study. Vaccine (pVAX/PSA) was administered in doses 100, 300 and 900 *μ*g (90% i.m. and 10% i.d.). Recombinant GM-CSF and IL-2 were administered s.c.

pVAX/PSA was given at three dose levels with three patients at each level (Figure 1

). The levels were determined arbitrarily by multiplying the dose by 3 as follows: 100 *μ*g given 90 *μ*g intramuscularly (i.m.) + 10 *μ*g intradermally (i.d.); 300 *μ*g − (270 *μ*g i.m. + 30 *μ*g i.d.) and 900 *μ*g − (810 *μ*g i.m. + 90 *μ*g i.d.). Vaccine was administered in the deltoid region for a total of five times at 4-week interval. To optimise vaccination, the following cytokines were given concomitantly as vaccine adjuvants: GM-CSF (Molgramostim, Leucomax®, Schering-Plough & Novartis, Sweden) (40 *μ*g day^−1^ × 3 days starting 2 days before vaccination) subcutaneously (s.c.) at the same site as vaccination, and IL-2 (Aldesleukin, Proleukin®, Chiron, Holland) (75 *μ*g day^−1^ × 7 days starting the day after vaccination) s.c. in the same extremity.

No chemotherapy, radiation therapy, corticosteroids (corresponding to >400 *μ*g day^−1^ of Bunesonide) or manipulation of anti-androgen suppression were allowed during the study.

All patients were evaluated for toxicity by the National Cancer Institute of Canada Clinical Trials Group (NCIC-CTG). Patients were enrolled in the higher dose cohort if dose-limiting toxicity ⩾3 was not reached.

### Vaccine (pVAX/PSA)

The vaccine was produced from a gene coding for the full-length human PSA protein (Lundwall and Lilja, 1987), obtained from Dr Tim, Ratliff, Washington University, St Louis, USA. It was inserted into the pVAX1 vector (Invitrogen, The Netherlands), a plasmid fulfilling the Food and Drug Administration, Center for Biologics Evaluation and Research (FDA CBER) regulations for vectors to be used in human DNA vaccination protocols. Sequence analysis revealed a single nucleotide difference of A to C at the position 289 compared with the published sequence (NM_001648).

The vaccine was produced by the ‘Gene Therapy Center’ at Huddinge Hospital, Stockholm, under Good Manufacturing Practice (GMP) conditions with endotoxin content ⩽10 EU mg^−1^,>85% supercoiled, protein content <10 *μ*g mg^−1^ of plasmid and chromosomal DNA content <30 *μ*g mg^−1^ of plasmid. The vaccine was aliquoted in saline solution, stored at −80°C and thawed just prior to administration. All handling of the DNA vaccine was performed according to NIH Guidelines for research involving recombinant DNA molecules. The PSA protein expression was verified by transfection into Cos7 cells and after i.m. injection in mice.

### Evaluation of immune response

Patient blood and serum were collected in heparinised containers and glass tubes, respectively, before the study, and 4 weeks after second and fifth vaccinations (Figure 1). Peripheral blood mononuclear cells (PBMC) were isolated by Ficoll density separation (Pharmacia, Uppsala, Sweden), resuspended in 90% autologous plasma and 10% DMSO, aliquoted and cryopreserved in liquid nitrogen until *in vitro* analysis. Patient serum was cryopreserved at −80°C until analysis.

#### Cellular response

Thawed PBMC were plated at 5 × 10^6^ ml^−1^ in 24-well plates and incubated for 2 h. Adherent cells were differentiated in X-vivo 15 medium with 2% autologous plasma supplemented with interleukin-4 (IL-4) (40ng ml^−1^) (Schering-Plough, Kenilworth, NJ, USA) and GM-CSF (50ng ml^−1^) (Schering-Plough & Novartis, Sweden). Immature dendritic cells (DC) were harvested on day 6 and loaded with recombinant PSA protein (Novavax Inc., Rockville, MD, USA). Briefly, Lipofectin (10 *μ*g) (Invitrogen, Carlsbad, CA, USA) and recombinant PSA (5 *μ*g) were mixed for 20 min at 20°C and thereafter added to DC in a total volume of 200 *μ*l X-vivo 15 medium and incubated for 4 h. DC were washed three times in PBS and treated with TNF*α* (50ng ml^−1^) (Chiron, Emmeryville, CA, USA) for an additional 48 h to allow maturation.

For stimulation, nonadherent cells, obtained from a second PBMC aliquot, were cultured with autologous PSA-transfected DC at a responder  stimulator ratio of 10 : 1 in X-vivo 15 supplemented with 2% autologous plasma, IL-7 (20ng ml^−1^) and IL-12 (100pg ml^−1^) (both R&D Systems Inc., Minneapolis, MN, USA) for 7 days. On day 8, responder cells were harvested, restimulated and resuspended in X-vivo 15 with 2% autologous plasma. At 2 days after restimulation, on day 10, IL-2 (R&D systems Inc.) was added to the culture at 20 U ml^−1^. On day 16 of culture, responder cells were harvested and analysed in duplicates for IFN*γ* production after 48 h coculture with PSA or human serum albumin- (HSA) (Pharmacia) loaded autologous DC (IFN-*γ* ELISA, Mabtech AB, Stockholm, Sweden). No other cytokines were tested.

#### Antibody response

Patient serum samples, collected before first vaccination and after five cycles of vaccinations, were analysed in parallel by ELISA for reactivity against recombinant PSA protein. Plates (96 well) were coated overnight at +4°C with human recombinant PSA protein at 2 *μ*g ml^−1^ in carbonate–bicarbonate buffer (pH 9.5). After blocking with PBS containing 0.05% Tween 20 and 0.25% bovine serum albumin (BSA), pH 7.4 (blocking buffer), serial dilutions of patient serum in blocking buffer were incubated for 4 h at room temperature. Secondary antibody peroxidase-labelled goat anti-human IgG (Southern Biotechnology Associated, Birmingham, AL, USA) was added. Assay was developed using DAKO TMB one-step substrate system (DAKO, Carpinteria, CA, USA) and measured spectrophotometrically at 450 nm. Samples were analysed in duplicate.

### Clinical monitoring

Each patient had a chest radiograph, bone scan and abdominal computer-assisted tomography (CAT) scan or magnetic resonance imaging (MRI), on entry and on week 20 (4 weeks after the fifth vaccination). Standard laboratory tests, before each vaccination cycle and 4 weeks after the last one, included: blood count, sedimentation rate, liver enzymes, creatinine, coagulation status, antinuclear and antimuscular antibodies, rheumatoid factor and PSA. PSA levels in patient plasma were measured using PROSTATUS™, fluoroimmunoassay (Wallac Oy, Turku, Finland) at the Department of Clinical Biochemistry, Karolinska Hospital. Patients were followed for minimum 16 weeks after the fifth vaccination or until inclusion in another study protocol.

## RESULTS

### Patient characteristics and treatment

Between August 2000 and January 2002, nine patients with histologically proven, PSA-positive, hormone-refractory prostate cancer were enrolled in this study. Their detailed characteristics are described in Table 1

**Table 1:** Characteristics of patients enrolled, including doses and adverse toxicities observed

**Patient #**	**Age (years)**	**Tumour grade^a^**	**Years since diagnosis**	**Months on hormonal therapy**	**ECOG status**	**Baseline PSA (ng ml^−1^)**	**Metastatic site**	**pVAX/PSA dose (*μ*g)**	**Adverse effects^b^ WHO grade**
1	64	2	7.4	20	0	15	None	100	2
2	80	3	6.8	45	1	39	Bone/lung	100	1
3	76	3	9.1	23	0	15	Bone	100	1
4	74	2	8.8	44	1	3.1	Bone/lung	300	1
5	71	2	6.3	68	0	15	Bone	300	1
6	68	Gleason score 4+4	9.5	10	0	9.2	Lymph node	300	Not evaluable^c^
7	68	2	3.3	40	0	11	None	900	1
8	78	2	5.5	13	1	9.2	None	900	1
9	62	2	10.0	48	1	3.6	None	900	2

ECOG=Eastern Cooperative Oncology Group; WHO=World Health Organization.

a Patients were diagnosed by fine-needle aspiration cytology and graded according to a three-tier system. In patient #6, the diagnosis was obtained using core-biopsy, reviewed and graded according to the Gleason system.

b Most common adverse toxicities were: systemic (reported in six patients) such as running nose, fatigue, myalgia, chills, and fever; and local at injection site (reported in seven patients) such as erythaema, swelling, induration, itching, local pain and papular urticaria.

c Patient developed upper urinary obstruction 1 week after first vaccination and was removed from study. This event was not related to investigational therapy.

. None of the patients received previous chemotherapy. Five had measurable metastasis and the other four had prostate cancer evaluable by increase in serum PSA. The study comprised five cycles of pVAX/PSA DNA vaccination in combination with recombinant GM-CSF and IL-2 protein administered as adjuvant (Figure 1). This adjuvant combination appeared beneficial compared to no adjuvant or either cytokine alone in a preclinical tumor-protection study (Roos *et al* (in press)). All patients had rising PSA on three consecutive occasions with on-study range between 3.1 and 39 *μ*g l^−1^. Their concurrent androgen-withdrawal therapy medication was not manipulated for 10 weeks before or during the study.

The dosage of the vaccine (100, 300 and 900 *μ*g) was determined arbitrarily; starting with the dose used in our preclinical toxicology studies submitted to the regulatory authorities (100 *μ*g). A threshold dose required to obtain significant responses was described at the time of initiation of the trial with malaria DNA vaccine in healthy volunteers and therefore the maximum dose was set at 900 *μ*g (Wang *et al*, 1998). The cytokine dose was kept constant in all cohorts in order to rule out the nonspecific stimulatory effect of the adjuvants.

Eight patients completed all five vaccination cycles and were fully evaluable for analysis. Patient #6 required withdrawal from study 1 week after the first vaccine administration due to bilateral upper urinary obstruction and hydronephrosis. This event was unrelated to the vaccination.

### Safety

The drug tested is not a cytotoxic agent, and therefore the study was not designed to determine the maximum tolerated dose (MTD), but to evaluate the safety and feasibility and to determine the biologically effective dose. No dose-limiting toxicity (DLT) occurred at any dose level tested. The vaccine-related toxicities are shown in Table 1. Neither the frequency nor the severity of adverse toxicities was associated with the administered dose of the vaccine. Treatment-related side effects did not exceed WHO grade 2. They were systemic, such as running nose, fatigue, myalgia, chills and fever (*n*=6), and at the injection site: erythaema, swelling, induration, itching, local pain and papular urticaria (*n*=7). Two patients, one receiving the lowest and one the highest vaccine dose developed the highest WHO grade 2 local reactions. The adverse toxicities at the injection site developed in association with administration of GM-CSF prior to pVAX/PSA administration. The systemic toxicities appeared several days post pVAX/PSA injection, during IL-2 administration. All adverse effects were self-limited within 2–3 days after the last IL-2 injection.

No significant changes occurred in the monitored serological parameters, except for minor elevation of antinuclear antibodies. The titre did not exceed 1 : 100. None of the patients developed clinically evident autoimmune disease.

### Responses

A secondary objective of this study was to determine immunological consequences of pVAX/PSA vaccination. For this reason, PBMC and serum samples were collected before the study, before the third vaccination and 4 weeks after the last vaccination (Figure 1). All cell and serum samples were frozen and stored at −180 and −80°C, respectively, until analysis. To minimise bias due to interassay variability, the analyses were performed simultaneously for all samples from each patient. The sample identity was blinded for evaluation. This approach did not influence the conduct of the trial, since the immunological efficacy of the vaccine was not critical for the continuation of the study.

#### PSA-specific cellular immune response

The cellular immune responses were assessed against the whole recombinant PSA protein for the following reasons: (1) this approach enables detection of immune responses elicited by both CD4+ and CD8+ T cells; (2) it prevents false-positive nonspecific reactivity against contaminants in the vaccine formulation and (3) in the published PSA vaccine trials (Meidenbauer *et al*, 2000; Heiser *et al*, 2002), immune responses were also monitored against full-length PSA protein thus enabling comparison.

In order to demonstrate PSA specificity of IFN-*γ* production, the nonspecific response against HSA was assayed in parallel for each sample

**Figure 2:**
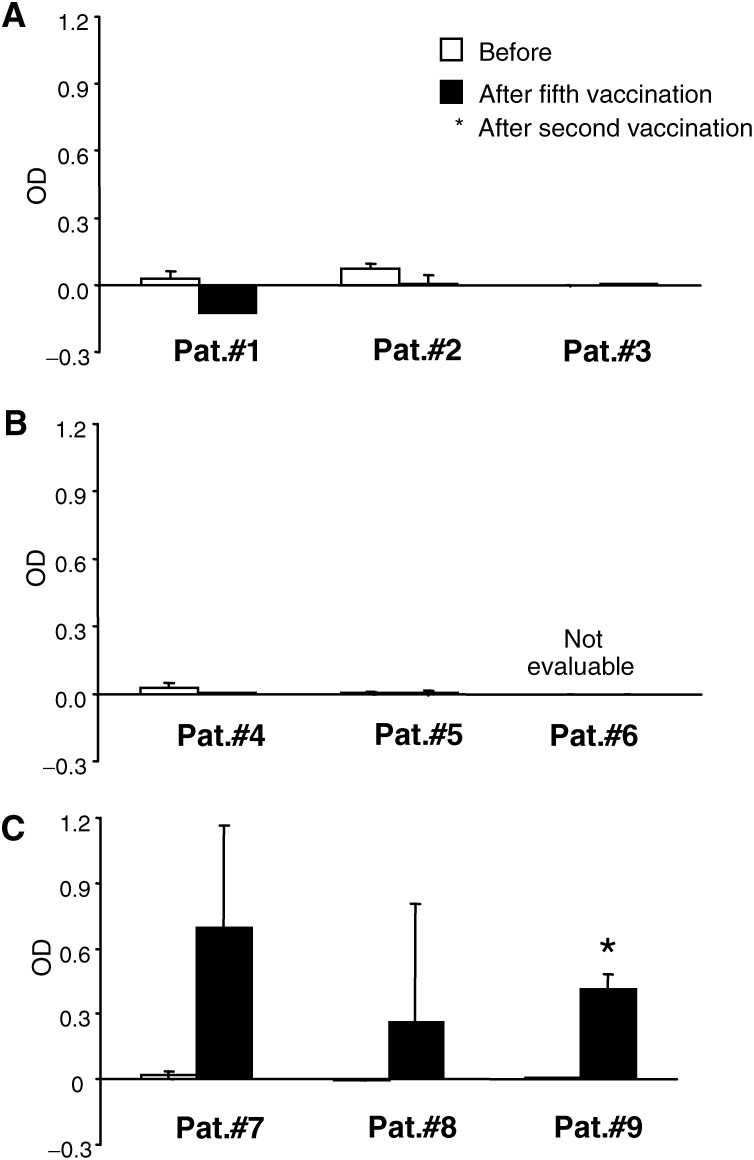
Induction of T-cell response to recombinant PSA, prior to and postvaccination, measured as IFN-*γ* production by ELISA after restimulation with DC loaded with PSA protein. Nonspecific reactivity against a control protein (HSA) has been subtracted from each individual sample. Patient groups vaccinated per cycle with 100 *μ*g (Pat#1–3), 300 *μ*g (Pat#4–6) and 900 *μ*g (Pat#7–9) of pVAX/PSA are shown in panels (**A**, **B** and **C**), respectively. Prior and post study values are presented if otherwise not stated. Bars represent mean values of two individual experiments with standard deviations.

No patient mounted a detectable PSA-specific IFN-*γ* response prior to vaccination (Figure 2

). Undetectable PSA-specific IFN-*γ* response was also present after vaccination in patient groups that received pVAX/PSA in the low and intermediate doses of 100 and 300 *μ*g (Figure 2A and B). Two of three patients that received the highest DNA vaccine dose (900 *μ*g) had a significant increase in IFN-*γ* response. In patient #8, the increase was not significant. Patient #7 had no detectable response after the second vaccination cycle (data not shown). The sample after the fifth vaccination is not available from patient #9 (Figure 2C).

#### PSA-specific antibody response

**Figure 3:**
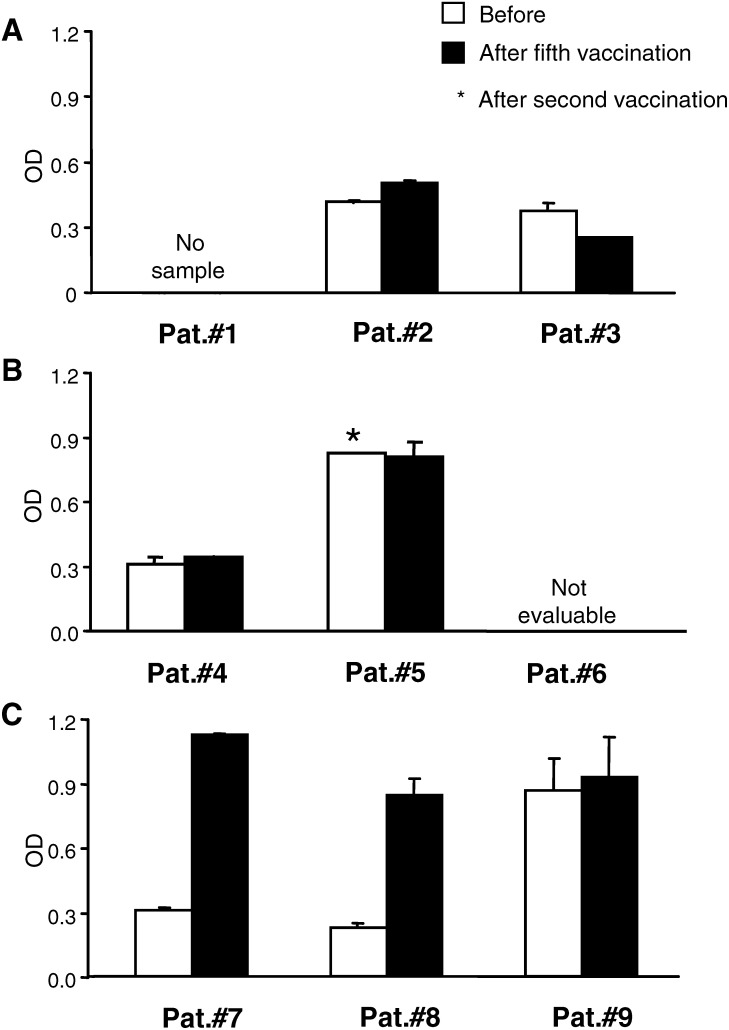
Induction of IgG antibody against recombinant PSA in serum measured at 1 : 100 dilution by ELISA prior to and post five cycles of vaccination. Patient groups vaccinated per cycle with 100 *μ*g (Pat#1–3), 300 *μ*g (Pat#4–6) and 900 *μ*g (Pat#7–9) of pVAX/PSA are shown in panels (**A**, **B** and **C**), respectively. Bars represent mean of duplicates with standard deviations.

Patient sera were also assessed for PSA-specific IgG levels before and after vaccination. While a PSA-specific cellular response was below detection limit in all patients prior to vaccination, certain patients exhibited low levels of PSA-reactive antibodies (Abs) at enrolment (Figure 3

). One exception was patient #9 who already had a significant anti-PSA level on presentation. In the low and intermediate dose group, no conclusive induction of PSA-specific Abs was detected (Figure 3A and B). In the highest dose group however, patient #7 and #8 developed a significant increase in anti-PSA Abs (Figure 3C). In patient #9, it remained an unchanged high, as compared to the prevaccination level.

#### Clinical consequences

**Figure 4:**
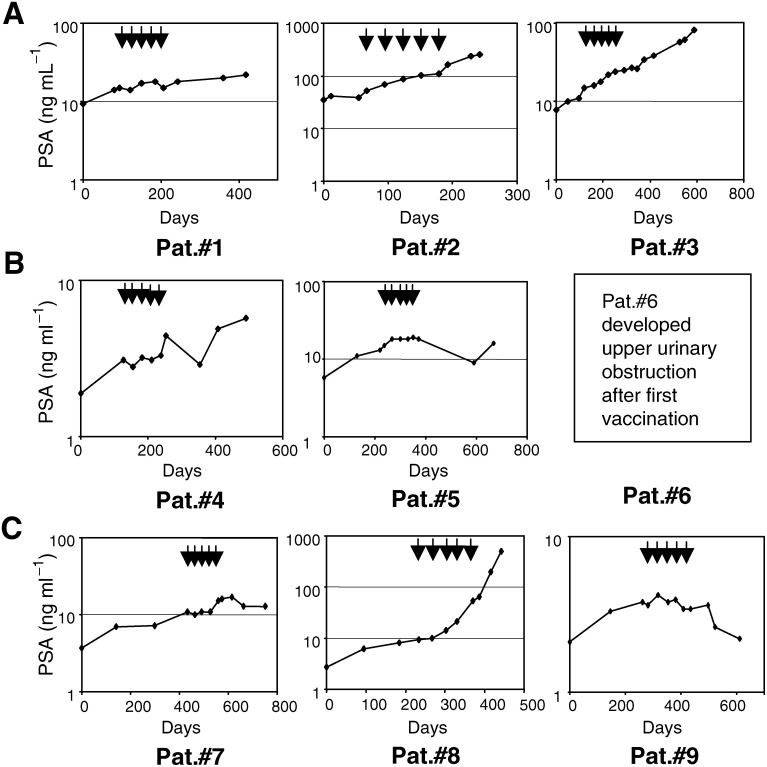
Prostate-specific antigen levels in patient serum measured by standard fluoroimmunoassay. Patient groups vaccinated per cycle with 100 *μ*g (Pat#1–3), 300 *μ*g (Pat#4–6) and 900 *μ*g (Pat#7–9) of pVAX/PSA are shown in panels (**A**, **B** and **C**), respectively. Arrows (↓) depict time of individual DNA vaccine administration.

The main objectives of this trial were to assess the feasibility, safety and immunogenicity of the DNA vaccine. Nevertheless, all enrolled patients were also considered for evidence of clinical responses. Patients #5, #7 and #9 developed an objective decrease in the slope of their PSA levels (Figure 4B and C

). All other patients (*n*=5) exhibited a biochemical progression with regard to their serum PSA levels (Figure 4). Four patients with evaluable metastasis on enrolment exhibited radiologically stable disease (no change in tumour volume). Patient #2 exhibited an objective progression of his bone metastasis as documented by the postvaccination bone scan. Patient #8 developed liver metastasis as determined by postvaccination CAT scan. Both of these events are a natural disease progression in this group of patients.

In conclusion, in this trial, a dose–response was observed with regard to induction of a PSA-specific immune response. The two patients that developed cellular immune response to PSA protein exhibited stabilisation of disease. In contrast, only one of six that did not develop PSA-reactivity showed clinical stabilisation. To demonstrate a significant antitumoral activity, a phase II trial needs to be performed at the highest dose of vaccine.

## DISCUSSION

This first clinical trial of a plasmid DNA vaccine encoding PSA was designed to evaluate the feasibility, safety and immunogenicity of pVAX/PSA in patients with prostate cancer. The novel finding of this trial is that this DNA/PSA vaccine, given together with GM-CSF and IL-2 to patients with prostate cancer, is safe and in doses of 900 *μ*g can induce PSA-specific immunity. As might have been expected from other clinical trials using DNA vaccines against human immunodeficiency virus (HIV), malaria, melanoma or B-cell lymphoma, only self-limiting adverse toxicities were observed with this pVAX/PSA vaccine (Calarota *et al*, 1998; MacGregor *et al*, 1998; Le *et al*, 2000; Timmerman *et al*, 2002; Rosenberg *et al*, 2003; Tagawa *et al*, 2003). The adverse toxicities observed in the trial were not associated with the dose of the vaccine administered, but time wise correlated with the administration of the two cytokines that were used as adjuvants.

The dose–response observed in this trial, with respect to the biologically effective dose, is in line with other trials with DNA vaccine and other antigens, in which a certain threshold was required to induce significant responses with DNA vaccination (Wang *et al*, 1998; Timmerman *et al*, 2002). Since there were no PSA-specific responses detected with the two lower DNA doses, the nonspecific enhancement of the immune status due to the administration of GM-CSF and IL-2 is unlikely. We are of the opinion that this effect is due to the increased amount of PSA protein produced. Nevertheless, it cannot be excluded that the higher doses of plasmid DNA itself might have functioned as immunological adjuvant. It is well established that bacterial DNA sequences, by virtue of different methylation pattern from mammalian DNA, may activate the immune system (Sato *et al*, 1996; Klinman *et al*, 1997).

It is interesting to note that a similar dose response was recently reported in rhesus macaques immunised with a similar DNA vaccine coding for human PSA (Kim *et al*, 2001). In this study, Kim *et al* observed PSA-specific induction of IFN-*γ* in animals that received 500 *μ*g or more of plasmid DNA. Our study extends these findings, demonstrating immune responses in patients exhibiting all impediments to immunotherapy associated with advanced stages of cancer and despite of the presence of large quantities of circulating self-antigen. Nevertheless, in stage IV melanoma patients vaccinated with 1 mg doses of plasmid DNA encoding gp100, Rosenberg *et al* (2003) have not detected any immune responses. The conclusions of this trial are uncertain as only five of the 22 enrolled patients completed the intended four cycles of vaccination, while others were taken off protocol after one or two cycles due to tumour progression.

The immunological monitoring is an important surrogate marker of all early-stage cancer vaccine trials, since other end points, such as patient survival in this advanced group of patients, are not indicative of vaccine potency. Nevertheless, the choice of the different monitoring tools is a matter of constant debate (Keilholz *et al*, 2002; Coulie and van der Bruggen, 2003). Although HLA A2-restricted PSA-derived peptides have been identified (Correale *et al*, 1997; Chakraborty *et al*, 2003), the use of peptides for immunological monitoring significantly limits the number of evaluable patients, when whole antigen vaccines are used. This was recently documented by Gulley *et al* (2002) in a trial with a recombinant vaccinia vector encoding PSA, where only five of the 42 enrolled patients could be immunologically tested.

Read-outs in this study were measured against recombinant PSA protein, to avoid nonspecific reactivity against contaminants in the vaccine formulation. By comparing the PSA-specific responses before and after vaccination, clonal amplification could be detected both in the T- and B-cell response. It is most unlikely, however, that the PSA-specific antibody responses are of clinical benefit, since PSA is a secreted antigen and thus this read-out serves merely as a marker of the effective delivery of the vaccine. It is therefore intriguing to note that antibody titres were highest after the vaccination in all three patients receiving 900 *μ*g doses, while only in one patient in the other two dosage cohorts. Furthermore, the T-cell response was enhanced in two of these patients.

The design of this study does not allow us to assess the value of the addition of GM-CSF and IL-2 as vaccine adjuvants nor the optimal vaccine dose or route of administration for induction of PSA-specific immune responses. The GM-CSF dose of 40 *μ*g was, per vaccination cycle, 30-fold lower compared to the dose of GM-CSF (Sangramostim) that modulated PSA kinetics when given as a single agent (Rini *et al*, 2003). The reduction in the slope of PSA increase observed in the two patients mounting cellular PSA responses is thus unlikely to be a GM-CSF effect. Antitumoral effects of IL-2 are limited in prostate cancer and otherwise only reported at higher treatment doses (Freedland *et al*, 2001)

Taken together, our study demonstrates the safety of a plasmid DNA vaccine encoding PSA, and that in doses of 900 *μ*g the vaccine can induce cellular and humoral immune responses against PSA protein. The proof for clinical benefit needs to be established in further trials. Nevertheless, these results provide the basis for further clinical development directed towards improving plasmid DNA vaccination procedures against solid tumours, which may lead to more potent and durable immunotherapeutical responses. The identification of the patient groups most likely to benefit from this immunotherapeutic treatment strategy provides a further challenge in future studies.
